# Size Distribution and Characteristics of Chitin Microgels Prepared via Emulsified Reverse-Micelles

**DOI:** 10.3390/ma12071160

**Published:** 2019-04-10

**Authors:** Siriporn Taokaew, Mitsumasa Ofuchi, Takaomi Kobayashi

**Affiliations:** Department of Materials Science and Technology, School of Engineering, Nagaoka University of Technology, 1603-1, Kamitomioka, Nagaoka, Niigata 940-2188, Japan; technomare3156@gmail.com (M.O.); takaomi@vos.nagaokaut.ac.jp (T.K.)

**Keywords:** crab shell, chitin, spherical microgels, reverse micelle, gelation

## Abstract

Chitin was extracted from local snow crab shell waste and used as a raw material in the fabrication of porous spherical microgels. The chitin microgels were obtained using a batch process of emulsification and, afterward, gelation. The effects of chitin concentrations, oil and water phase ratios (O:W), surfactants, and gelation on the size distribution and morphology of the microgels were investigated. The extracted chitin possessed α-chitin with a degree of acetylation of ~60% and crystallinity of 70%, as confirmed by Fourier Transform Infrared Spectroscopy (FTIR) and X-Ray Powder Diffraction (XRD). In the reverse-micellar emulsification, different chitin concentrations in NaOH solution were used as aqueous phases, and n-hexane media containing Span 80-based surfactants were used as dispersion phases. Various HCl solutions were used as gelling agents. Microgels with sizes ranging from ~5–200 μm were obtained relying on these studied parameters. Under the condition of 3% *w*/*w* chitin solution using O:W of 15:1 at 5% *w*/*w* of Span 80 (hydrophilic-lipophilic balance; HLB of 4.3), the gelation in the emulsified reverse micelles was better controlled and capable of forming spherical microgel particles with a size of 7.1 ± 0.3 μm, when 800 μL of 1 M HCl was added. The prepared chitin microgel exhibited macro-pore morphology and swelling behavior sensitive to the acidic pH.

## 1. Introduction

Chitin (*ß*-(1,4)-*N*-acetyl-D-glucosamine) is a natural polysaccharide found in shells of marine animals such as shrimp, lobster, and crabs. As a marine fishery product, about 30 thousand tons of crabs were annually caught in Japan [1]. This means that large amounts of crab shells were disposed in landfills without being recycled. It is known that crab shells are a good biomass source of chitin [2]. Due to biocompatibility and non-allergenicity, chitin has been widely used in pharmaceutics and bio-medical drugs. In addition, chitin extracted from crab shells has other characteristics such as antibacterial properties and protein affinity that are useful for wound dressing and controlled-drug release applications [3,4]. As a drug carrier, small spheres of gel forming chitin have been recognized as having high drug loading capacity, efficient drug control at the target site, sustained drug release, and high stability compared to micelles and lipid-based carriers [5,6]. Moreover, microgels can be applied as adsorbents, chemical/biological sensors, enzyme immobilization, and gene delivery vehicles [5,7]. 

The applications of chitin and its derivatives as microgels/microparticles were reported as biological filling [8] and drug delivery agents [6]. Chitin microparticles could regulate the depletion of cholesterol by cellular macrophage activation [9]. A fragmented physical hydrogel suspension of chitin derivatives was indicated to support reepithelization of spinal tissue and vasculature with minimal fibrous glial scaring [8]. Moreover, the fragmented chitin microgels loaded with an anti-metabolite drug for delivery in psoriasis treatment exhibited higher skin permeable efficacy than those of the control drug solution and the conventional drug gel. The drug-loaded fragmented chitin microgels also exhibited greater swelling and drug release at acidic pH than in neutral and alkaline conditions [6]. However, the time window of the use of microgels with an unspecific-shape was difficult to determine with high precision because the shape and the stimuli responsiveness influence the biodistribution, the circulation dynamics, the drug release, and the intracellular uptake of the microgels [8,10]. Hence, the fabrication of microgels that have precise geometries and stimuli-responsiveness has been significant in particle transportation and therapeutic agent delivery. 

It has been reported that several microgel preparation methods including solid-phase organic synthesis [11], microfluidics [12], and emulsification [13,14] were feasible. However, solid-phase organic synthesis and microfluidics have numerous problems associated with the use of cross-linked insoluble polymers, the fluctuation of reaction rates, and the longer time-consumption in such processes [15,16]. In contrast, a reverse-micellar emulsification technique simplifies the process, making it an effective tool to synthesize small particles with controllable size and shape. An emulsion-based method is also energy-efficient, non-destructive, and attractive for large-scale production [13,14]. As compared to the other approaches, the reverse-micellar emulsification can enhance uniformity and dispersity of the polymeric particles, can be operated at low temperature, and provide a stable dispersion for a water in oil emulsion system [17]. Therefore, such a method is used to prompt self-assembly of surfactant in organic media, whereby the oil region having a nonpolar nature faces the outside surface of the micelle, and the polar region forms the core for polymeric microgels [18]. In such a structure, the tiny aqueous droplets with varied sizes are encapsulated, and the different-sized microgels are produced within the reverse micelle after gelation [13]. Accordingly, chitin microgels with the same size prepared by the reverse-micellar emulsification method can be described. 

The aim of the present study was to prepare chitin microgel by using a reverse micelle system at various compositions of water in oil (W/O) emulsions. The chitin used in this study was extracted from shell waste of red snow crabs, which was collected from the local area in Niigata prefecture, Japan. The extracted chitin was then characterized and compared to commercial chitin and chitosan. The synthesis of the microgel was performed using a batch process of W/O emulsion. The effects of chitin concentrations (water phase) of 1–3% *w*/*w* and oil:water phase ratios of 3–15:1 were studied. It was known that hydrophilic-lipophilic balance (HLB) values of surfactant ranging from 3.5 to 6 were more suitable for a W/O emulsion system [19]. For Span 80, a nonionic-based surfactant, the HLB value of 4.3 was adjusted to 5 and 6 in this study. Concentrations of Span 80 (3–7% *w*/*w*) containing n-hexane (oil phase), and gelation using HCl were also investigated in terms of their size distribution and morphology.

## 2. Materials and Methods 

### 2.1. Materials

Dried, cleaned snow crab shells, *Chionoecetes opilio*, were obtained from Teradomari port, Teradomari, Niigata prefecture, Japan. Chemicals were purchased from Wako Pure Chemical Industries, Ltd, Osaka, Japan. Distilled and ion-exchanged water was used in all the experiments.

### 2.2. Extraction of Chitin

The coarse flakes of crab shells (30 g) were hydrolyzed using 900 mL of 1.0 M HCl under stirring at room temperature (20 ± 5 °C) for 24 h. The reaction was stopped by adding water and filtered through a mesh sieve to remove small contaminants. Protein residuals were removed by heating the hydrolyzed chitin at 90 °C in 900 mL of 1.0 M NaOH under stirring for 5 h. Pigments in chitin were removed by stirring in 900 mL of ethanol for 5 h at 60 °C. The extracted chitin was dried in vacuum oven at 60 °C for 24 h, and ground in a blender.

### 2.3. Preparation of Microgels

**Figure 1:**
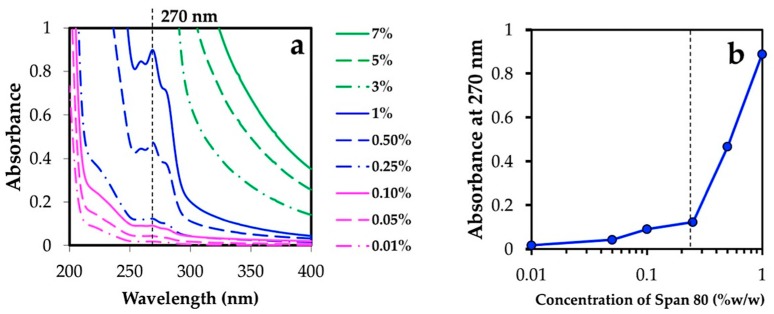
UV-Visible absorbance versus concentration profile of Span 80 in n-hexane at 20 ± 5 °C (**a**), and absorbance at 270 nm of Span 80 at various concentrations (**b**). Span 80 was dissolved in n-hexane at a concentration of 0.01–7% *w*/*w*. After thorough mixing, the solution was transferred to a 1.0 cm quartz cell and the spectrum was recorded at wavelengths of 200–400 nm using UV-visible near-infrared spectrophotometer (Jasco V570, Jasco Corporation, Tokyo, Japan). Blank n-hexane was used as a reference. The vertical dashed line in (**b**) marks the critical micelle concentration.

The chitin powder was dissolved in 20% *w*/*v* NaOH at −20 °C under periodic stirring to obtain 1, 2, and 3 % *w*/*w* of chitin aqueous solution. Before emulsion formation, the oil layer solution consisted of n-hexane and surfactants were used as a dispersion phase and prepared in a 50 mL amber vial. According to the critical micelle concentration (CMC) of Span 80 in n-hexane, see Figure 1, below the CMC in the presence of surfactant monomer, there was no peak throughout the spectrum, see Figure 1a, but the peaks at 270 nm appeared at above the CMC. The CMC was approximately 0.25% *w*/*w*, determined by the change of the trend in Figure 1b, which indicates the initial formation of micelles. Above the CMC value (about 10 times), a number of surfactant molecules were able to gather and form stable micelles in the bulk liquid [20]. Therefore, 3, 5, and 7 % *w*/*w* of Span 80 were adopted for this study. A Span 80 (Sorbitan monooleate)-based surfactant was mixed with sodium cholate (HLB 18) to obtain HLB values of 4.3, 5, and 6. The chitin solution was dropped into the dispersion phase with vigorous stirring at 1500 rpm at room temperature (20 ± 5 °C) for 45 min. The W/O emulsion was then heated to 65 °C. Aqueous HCl solution in the range of 0.01–0.1 M concentration was used as counter-ions. In the gelation process of chitin microgel, 400–1200 µL of aqueous HCl solution was periodically dropped into the emulsion under stirring at 150 rpm. The parameters tested in the preparation of microgels are shown in Table 1. The chitin microgels were coagulated in the liquid medium and precipitated. The microgel was purified to remove the surfactant and residual n-hexane using dialysis (Molecular weight cut-off of 12 kDa, 0.5 nm, AS ONE corporation, Osaka Japan) in 1L of distilled deionized water for 72 h. 

### 2.4. Characterization

#### 2.4.1. X-Ray Fluorescence Spectroscopy (XRF)

An elemental study of the extracted chitin was performed using an X-ray fluorescence spectrometer (Rigaku ZSX Primus II, Tokyo, Japan) using ZSX software. This spectrometer contains a 50 keV and 50 mA X-ray tube, providing the detection of diverse elements of the Periodic Table. Prior to characterization, the sample pellets were prepared by using pressed powder method under a pressure of 500 kgf/cm^2^. 

#### 2.4.2. X-Ray Powder Diffraction (XRD)

X-ray diffractograms were obtained using an X-ray diffractometer (Rigaku Smart Lab 3 kW, Tokyo, Japan) under operation conditions of 40 kV and 30 mA with Cu Kα radiation. The relative intensity was recorded in steps of 0.1° and at a speed of 3.0 °/min. The crystallinity index (*CrI*) was determined by integrated X-ray powder diffraction software (Rigaku PDXL2, Rigaku Corporation, Tokyo, Japan). The quantitative analysis was performed based on the Rietveld refinement and an ab-initio crystal structure determination using crystal structure information of α-chitin provided by the software. The degree of acetylation (*DA*) of chitin [21] was calculated by:

(1) $$DA\;\left(\%\right)=100-\frac{\left(103.97-CrI\right)}{0.7529}$$

#### 2.4.3. Fourier Transform Infrared Spectroscopy (FTIR)

FTIR spectra were obtained using a FTIR spectrometer (Jasco 4100, Jasco Corporation, Tokyo, Japan). The sample pellets were prepared using the KBr method. The absorption bands were scanned between 4000–400 cm^−1^. The degree of acetylation (DA) was calculated by:

(2) $$DA\;\left(\%\right)=\frac{1}{1.33}\left(\frac{A_{1655}}{A_{3450}}\right)\times 100$$

where *A*_1625_ and *A*_3450_ are values of absorbance measured at 1625 and 3450 cm^−1^, respectively [22].

#### 2.4.4. Differential Scanning Calorimetry (DSC)

Thermograms were carried out using differential scanning calorimetry (DSC) (Rigaku, Thermo Plus EVO DSC823, Tokyo, Japan) under an air atmosphere. Dried samples (3–5 mg) were placed in hermetically sealed Al pans and immediately loaded in the DSC chamber. A sealed empty pan was used as a reference. Samples were scanned at the heating rate of 5 °C/min through the temperature range of 50–400 °C.

#### 2.4.5. Dynamic Light Scattering (DLS)

The size distributions of the microgel samples subjected to the tested parameters and swelling test at different pH values of 2, 4, 7, and 10 were analyzed by dynamic light scattering (DLS Shimadzu SALD-7000, Tokyo, Japan). pH values in the swelling test were adjusted by using 0.1 M HCl and 0.1 M NaOH. 

#### 2.4.6. Optical Microscopy, Scanning Electron Microscopy (SEM), and Transmission Electron Microscopy (TEM)

The optical microscopic morphologies of the reverse micelles in the emulsion and the microgels were visualized using an optical microscope (Olympus CKX41 Inverted Phase Contrast Microscope, Tokyo, Japan) at the magnification of 20×. Morphologies of the freeze-dried microgels were studied by scanning electron microscopy (Desktop SEM Hitachi TM3030 Plus, Tokyo, Japan) and transmission electron microscopy (TEM Hitachi HT7700, Tokyo, Japan). The microgels were freeze-dried by immersing in liquid N_2_ for 1 h before immediately loading the frozen samples into a chamber of a freeze dryer (Eyela Freeze Dryer FDU-1200, Tokyo, Japan). The freeze-drying process was operated at a condenser temperature of −40 °C under high vacuum. For SEM, the freeze-dried microgels were coated with gold using a gold sputter (Quick cool coater SC-701MC, Tokyo, Japan) under a high-vacuum condition. The surface morphology of the coated microgels was then observed at a voltage of 15 kV using a back-scatter detector (BSE) mode at 2000×. For TEM, the freeze-dried microgels were stained with Osmium tetroxide for 20 s before observing the sample morphology at 100 kV and 4000×.

#### 2.4.7. Zeta-Potential

Zeta-potential of the microgels was determined using a zeta-potential analyzer equipped with auto-titrator, stirrer, and inbuilt peristatic pump (Otsuka ELSZ, Tokyo, Japan). The zeta-potential was recorded at the pH values ranging from 2 to 10 adjusted using 0.1 M HCl and 0.1 M NaOH. All measurements were carried out at room temperature (20 ± 5 °C).

#### 2.4.8. Brunauer-Emmett-Teller (BET)

N_2_ adsorption-desorption isotherms of freeze-dried chitin microgels were carried out using a surface area and porosity analyzer (Micromeritics TriStar II, Norcross, GA, USA) at 77 K using Brunauer-Emmett-Teller (BET) and Barrett-Joyner-Halenda (BJH) analyses. Before analysis, samples were degassed at 30 °C on a vacuum line for 24 h.

## 3. Results and Discussion

### 3.1. Properties of the Extracted Chitin

Chitin extraction, in this work, included acid-base hydrolysis and decoloration processes. The crab shell waste was extracted for chitin having yield of 25 ± 8% dry weight. 

From XRF analysis, see Table 2, the extracted chitin retained high contents of C and O of the organic compound. The other sea contaminants in the extracted chitin were mainly removed from the crab shells and the quality was similar to the commercial chitin. While the heavy metals in the extracted chitin were not detected as compared to chitin from red shrimp shell [23].

**Figure 2:**
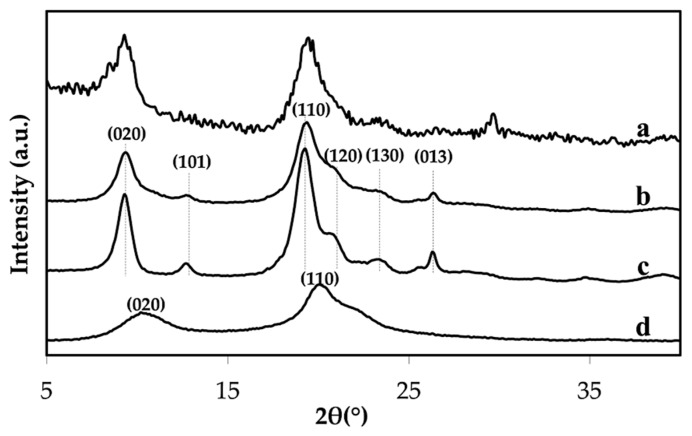
X-ray diffractograms of crab shell (**a**), extracted chitin (**b**), commercial chitin (**c**), and commercial chitosan (**d**).

Figure 2 shows XRD patterns of the extracted chitin obtained in the 2*θ* range of 5–40°. The diffraction peaks of the extracted chitin, see Figure 2a, and the commercial chitin, see Figure 2b, at 9.4°, 12.8°, 19.4°, 20.8°, 23.5°, and 26.4° were observed with indices of (020), (101), (110), (120), (130), and (013). These parameters define the crystallographic planes of α-chitin. This indicated that chitin has high molecular packing with inter- or intramolecular hydrogen bonds, imparting a high degree of crystallinity [23,24,25]. The intensities of the (020) and (110) planes decreased and moved to higher angles with a reduction in the degree of acetylation (DA) [21]. In this work, characteristic peaks of chitosan indexed as (020) and (110) appear at 10.4 and 20.3°, respectively, see Figure 2d. The extracted chitin exhibited a crystallinity value of 70.3%, as shown in Table 3. The DA of the extracted chitin obtained by XRD and confirmed by FTIR techniques were 55.3% and 60.9%, respectively, see Table 3. This meant that the extracted chitin was partially deacetylated. 

**Figure 3:**
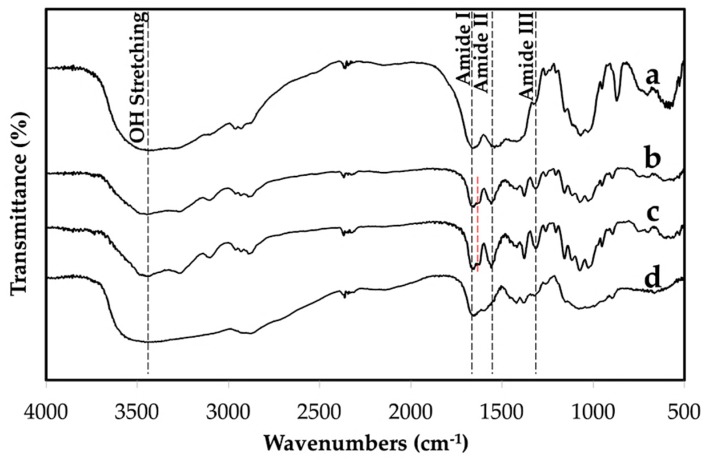
Fourier transform infrared (FTIR) spectra of crab shell (**a**), extracted chitin (**b**), commercial chitin (**c**), and commercial chitosan (**d**).

**Figure 4:**
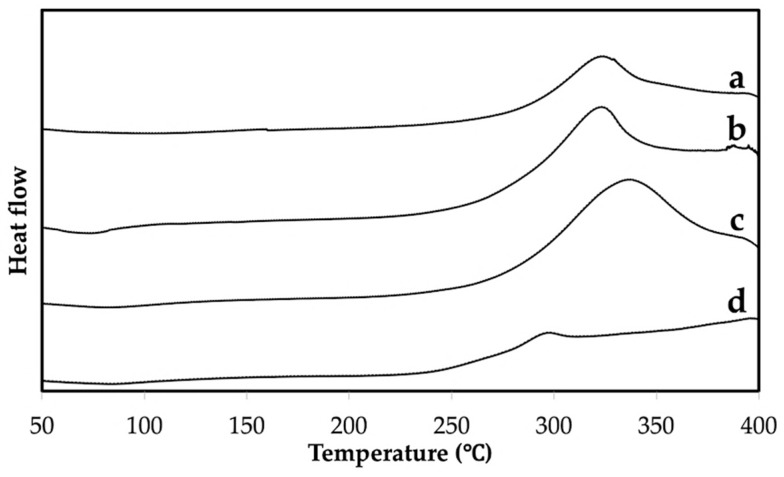
Differential scanning calorimetry (DSC) thermogram of crab shell (**a**), extracted chitin (**b**), commercial chitin (**c**), and commercial chitosan (**d**).

As seen in Figure 3, the FTIR spectrum of the extracted chitin had a broad peak at about 3450 cm^−1^ assigned to OH stretching. Amide I, II, and III appeared at the observed absorption bands around 1652, 1557, and 1310 cm^−1^, respectively. It was observed that the amide I band of the extracted chitin is split into two 1652 and 1623 cm^−1^. The existence of these interchain bonds of carbonyl groups of amide I and II are responsible for the high chemical stability of the α-chitin structure [23,26]. DSC thermograms of the crab shell, extracted chitin, commercial chitin, and chitosan were compared, see Figure 4. The wide and weak endothermic peak of the extracted chitin in Figure 4b was noticed at about 50–90 °C and ascribed to the loss of bound water. The exothermic peak of the crab shell and extracted chitin was observed at 330 °C due to the crystalline α-chitin structure. This indicated that the extraction process of chitin retained the α-structure of the resulting product. The extracted chitin had a higher temperature at which the exothermic peak appeared than the chitosan, see Figure 4d. The exothermic peak observed for chitosan at 295 °C is the characteristic peak of amine (GlcN) unit decomposition [27]. 

### 3.2. Reverse Micelle Emulsification for the Fabrication of Chitin Microgels

#### 3.2.1. Effect of Water, Oil Phase, and Surfactant

**Figure 5:**
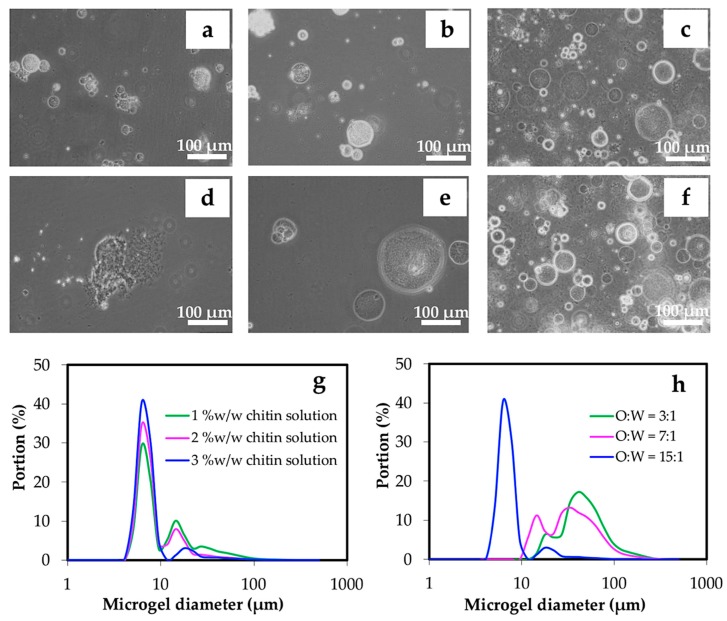
Optical microscopic images of microgels prepared from chitin solution at the concentrations of 1% *w*/*w* (**a**), 2% *w*/*w* (**b**), and 3% *w*/*w* (**c**), and O:W of 3:1 (**d**), 7:1 (**e**), and 15:1 (**f**) by controlling the concentration of Span 80 (HLB 4.3) at 5% *w*/*w* in oil phase. Gelation was carried out using 800 μL of 1.0 M HCl. The representative size distributions of microgels prepared by different chitin concentrations and O:W ratios are shown in (**g**) and (**h**), respectively.

In the reverse micelle emulsification, chitin in the alkali solution was prepared at 1, 2, and 3 % *w*/*w*, and then added dropwise into the oil phase. The microgels produced from 1 and 2 % *w*/*w* of the chitin solutions became small in size, but rather aggregated, see Figure 5a,b.

Increasing the chitin concentration to 3% provided more dispersed microgels with an average size of 7.1 ± 0.3 μm, see Figure 5c,g. Nevertheless, microgels produced from 3% chitin appeared as a weak gel with a less uniform size, as seen in Figure 5d,e. These differences are due to the low ratios of oil and water phases (O:W) at 3:1 and 7:1. At O:W of 15:1, the microgel appeared to be more dispersed, see Figure 5f. From the dynamic light scattering analysis, chitin microgels prepared from 1–3% *w*/*w* of chitin and O:W of 15:1 yielded a narrower size distribution (5–10 μm), see Figure 5g. However, there were wider size distributions (10–100 μm) of microgels when O:W of 3:1 and 7:1 were used (Figure 5h). Due to the low O:W of 3:1 and 7:1, the reverse micelles of chitin could not properly disperse in the oil phase during agitation. This might be due to the bigger microgels yielded after gelation, meaning that, low volume of the dispersion phase caused a high incidence of micelle breaking collisions during agitation.

**Figure 6:**
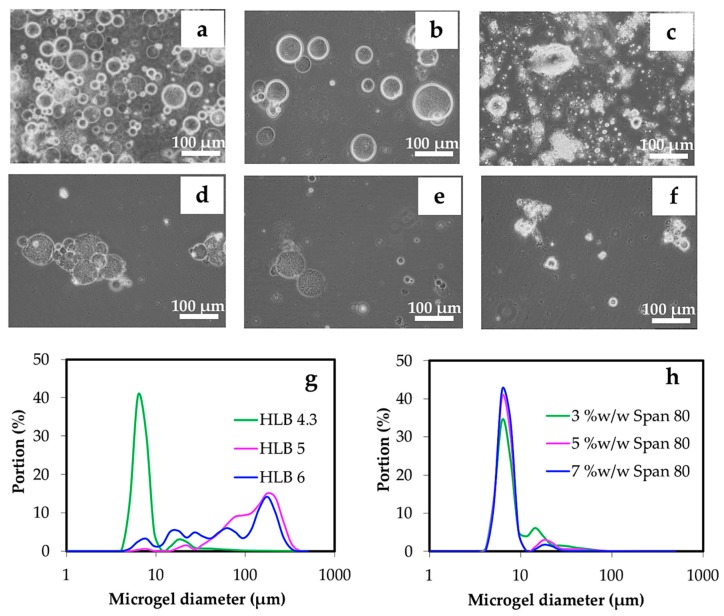
Optical microscopic images of microgels prepared from hydrophilic-lipophilic balance (HLB) values of surfactant at 4.3 (**a**), 5 (**b**), and 6 (**c**), and Span 80 at the concentrations of 3 % *w*/*w* (**d**), 5 % *w*/*w* (**e**), and 7 % *w*/*w* (**f**) by controlling the concentration of the chitin solution at 3 % *w*/*w* and an O:W ratio at 15:1. Gelation was carried out using 800 μL of 1.0 M HCl. The representative size distributions of microgels prepared by different HLB values and Span 80 concentrations are shown in (**g**) and (**h**), respectively.

It can be clearly observed in Figure 6a,g that HLB 4.3 was suitable for preparing chitin microgels in this study as compared to mixed surfactants having HLB 5 and 6 due to the balance of the size and strength of hydrophilic and lipophilic moieties of surfactant molecules. The bigger microgels with wider size distribution were the result of using mixed surfactants at HLB of 5 and 6, see Figure 6b,c,g. This was due to the higher hydrophilic portion in the surfactant that allowed the chitin aqueous solution to form larger and stable cores inside the reverse micelles. 

Span 80 concentrations (HLB 4.3) of 3, 5, and 7 % *w*/*w* were varied in the preparation of chitin microgels. In this range of surfactant concentrations, the morphology of the resulting microgels observed through the optical microscope were similar in size, see Figure 6d–f. The size distributions were also comparable, when the Span 80 concentrations were in the range of 3–7% *w*/*w*, see Figure 6h. However, as seen in Figure 6, the microgel prepared under the condition of 3% *w*/*w* surfactant likely exhibited aggregation, in which slightly a larger portion of ~20 μm microgel was observed.

#### 3.2.2. Effect of Gelation

**Figure 7:**
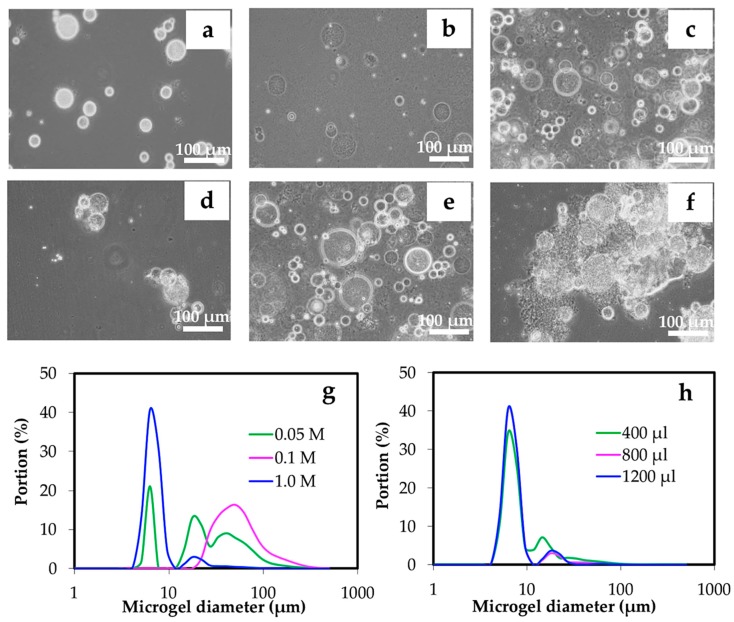
Optical microscopic images of microgels prepared from gelation using HCl at the concentrations of 0.05 M (**a**), 0.1 M (**b**), and 1.0 M (**c**), and 400 μL (**d**), 800 μL (**e**), and 1200 μL (**f**) of 1.0 M HCl by controlling the concentration of chitin solution of 3% *w*/*w* at an O:W ratio of 15:1, and concentration of Span 80 (HLB 4.3) at 5% *w*/*w* in the oil phase. The representative size distributions of microgels prepared by different concentrations and volume of HCl are shown in (**g**) and (**h**), respectively.

The gelation was implemented while the alkali chitin solution inside the emulsified reverse micelles was surrounded by an oil phase. When HCl solution was used as the gelling agent, the alkali conditions of the chitin solution have a neutralizing acid-base reaction. From Figure 7a–c,g, the concentration of HCl greatly affected the size distribution. When the HCl concentration was changed to 0.05, 0.1, and 1 M, a size variation (~5, 20, and 40 μm) was observed as the diluted HCl concentration was 0.05 M. It was seen that the size was less variant with increased HCl concentrations. As seen in Figure 7g, concentrations of 0.1 M and 1.0 M provided 50 and 5 μm diameter microgel spheres, respectively. This was related to the effect of the water phase on the microgel preparation, in which the initial concentration of chitin solution and external water affected the size distribution of the microgels. It was possibly owing to the involvement of a higher amount of water in the emulsion system; Span 80 was able to hold/absorb water into the core of reverse micelles [14]. The effect of a volume of 1.0 M HCl was further studied, see Figure 7d–f,h. It was found that only 400 μL was sufficient to yield the narrow size distribution of ~5 μm microgels. However, increasing the volume to 800–1200 μL slightly increased the portion of ~5 μm microgels. 

### 3.3. Properties of Chitin Microgels Prepared by the Reverse Micellar Method

**Figure 8:**
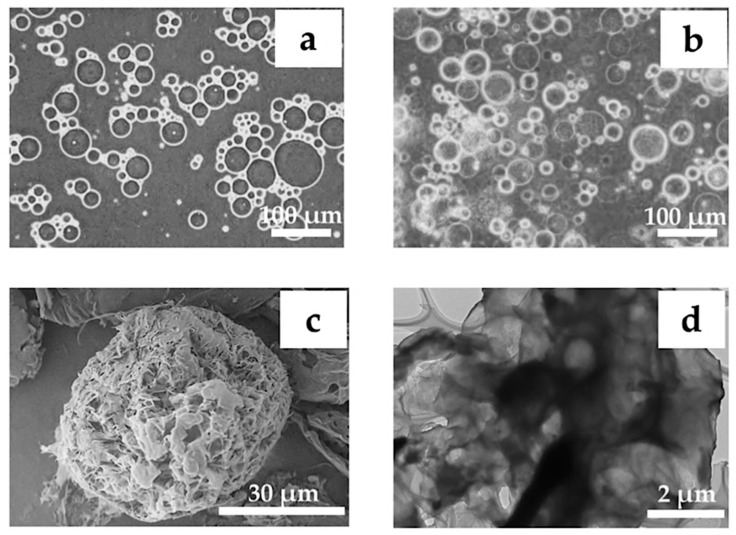
Optical microscopic images of reverse micelles in emulsion (**a**) and microgels (**b**) prepared under 3 % *w*/*w* chitin solution, O:W = 15:1, 5% *w*/*w* Span 80 (HLB 4.3), and gelation by 800 μL of 1.0 M HCl. Morphologies of the freeze-dried microgels are shown at magnifications of 2000× by scanning electron microscopy (SEM) (**c**) and 4000× by transmission electron microscopy (TEM) (**d**).

The appearance of chitin solution containing reverse micelles is shown in Figure 8a, corresponding to microgels prepared with 3% *w*/*w* chitin solution, O:W of 15:1, 5% *w*/*w* Span 80 (HLB 4.3), and gelation by 800 μL of 1 M HCl. As compared to the size of reverse micelles, no remarkable change in size was observed after gelation of chitin microgels. Figure 8b showed no microgel breakage induced by collision during stirring. Electron microscopic images of the freeze-dried samples revealed a spherical chitin microgel with macropores on the surface, see Figure 8c, and an internal porous structure, see Figure 8d. The formation mechanism of macropores on microspheres prepared by an emulsion system when using Span 80 for the Poly(styrene-divinyl benzene) system has been reported [14]. Similarly, macropores of the resultant chitin microgels are strongly related to the absorption of water from the external aqueous phase into the reverse micelles. Since Span 80 with a HLB of 4.3 was less hydrophobic, it has a stronger ability to absorb water. 

**Figure 9:**
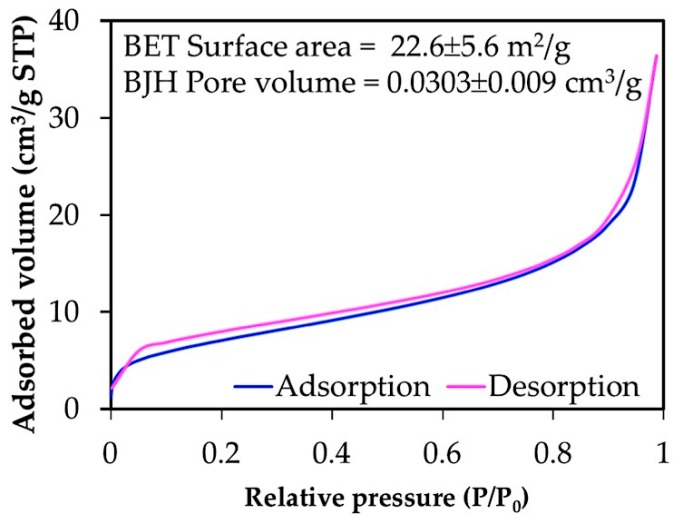
Brunauer-Emmett-Teller (BET) isotherm of freeze-dried microgels prepared under conditions of 3 % *w*/*w* chitin solution, O:W = 15:1, 5% *w*/*w* Span 80 (HLB 4.3), and gelation by 800 μl of 1.0 M HCl.

The porosity data of the chitin microgels was characterized by BET. The BET isotherms of the microgels are shown in Figure 9. The chitin microgels showed a type II isotherm for a microporous material according to the IUPAC classification [28]. The surface area and pore volume were 22.6 m^2^/g and 0.03 cm^3^/g, respectively.

**Figure 10:**
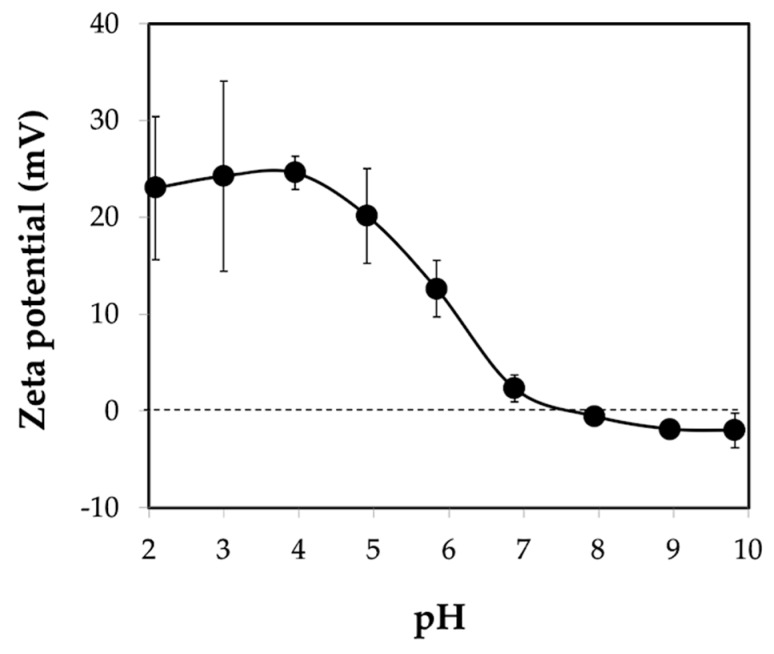
Zeta potential of chitin microgels prepared under conditions of 3 % *w*/*w* chitin solution, O:W = 15:1, 5% *w*/*w* Span 80 (HLB 4.3), and gelation by using 800 μL of 1.0 M HCl.

Under the optimal conditions of microgel preparation, the charge of partially deacetylated chitin microgels was investigated by measuring the zeta potential according to various pHs. Figure 10 shows the zeta potential of the chitin microgels prepared at 3% *w*/*w* chitin solution, O:W of 15:1, 5% *w*/*w* Span 80 (HLB 4.3), and gelation by 800 μL of 1.0 M HCl. The positive zeta potential at pH below 6 clearly indicated partially deacetylated chitin with different %DA due to the different number of amine groups to be protonated, leading to the positive charges. The isoelectric point (IP), that is the null zeta potential, increases to a higher pH value with lower %DA. Accordingly, IP values of chitosan (DA < 50%) and chitin (DA > 50%) were detected at pH values of 8.2–8.8 and 7.3–7.6, respectively [24,29]. In the present work, the IP value of the chitin microgels was observed at about pH 7.6, see Figure 10. This confirms that the chitin microgels were not further deacetylated during the preparation process of the chitin microgels. 

**Figure 11:**
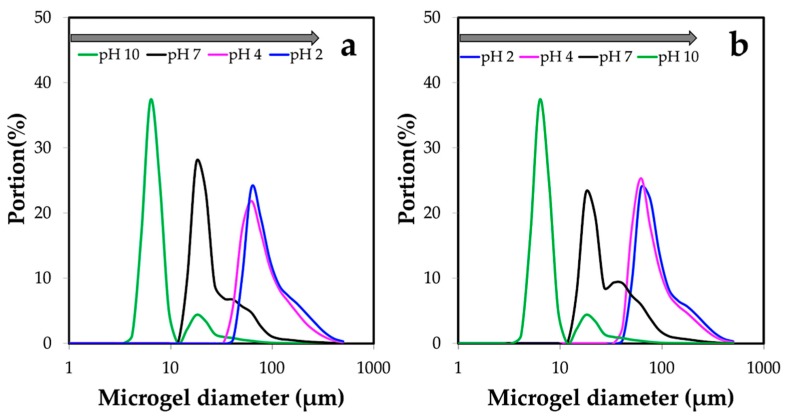
Reversible swelling-shrinking behavior of chitin microgels prepared under conditions of 3 % *w*/*w* chitin solution, O:W = 15:1, 5% *w*/*w* Span 80 (HLB 4.3), and gelation by using 800 μL of 1.0 M HCl. Responsiveness to pH was tested from pH 10 towards pH 2 (**a**) and pH 2 towards pH 10 (**b**).

Since the microgels presented pH dependence, it showed different swelling of the microgels tested over the entire range of pH from 10 to 2. From Figure 11a, the chitin microgels swelled at low pH (pH < IP). Microgels that were approximately 6 μm in size increased to ~60 μm. However, the gradual decrease was observed between pH 2 and pH 4 due to the similar zeta potential of approximately +25 mV, as seen in Figure 10. This was possibly the impact of the degree of acetylation, which controls the characteristics and activities of chitin [30]. In the extracted chitin with a moderate degree of acetylation (~60%), a number of amino groups in the chitin polymer chains were protonated while exposed to a specific pH. The protonation leads to the repulsion of polymer chains and allows more water to enter into the microgel network; consequently, swelling occurs [31]. After adjusting the pH backward, from 2 to 10, a reversible swelling-shrinking behavior was noticed. The chitin microgels began to shrink to a smaller size as the pH increased (pH > IP), see Figure 11b. This was because the deprotonation made the electrostatic interactions in the microgel network reconstruct [31]. 

## 4. Conclusions

Chitin extracted from crab shell waste was used for microgel fabrication. Simple gelation inside the emulsified reverse-micelles with low energy consumption was applied to prepare the chitin microgels. The spherical size distribution and the morphology of the microgels were greatly affected by the volume of the dispersion phase, hydrophilic-lipophilic balance of the used Span 80 surfactant, and concentration of the gelation agent. As a result, the chitin microgel with narrow size distribution (average size of 7.1 ± 0.3 μm) and porous spherical morphology was achieved under the condition of 3 % *w*/*w* chitin solution, O:W of 15:1, 5% *w*/*w* Span 80 (HLB 4.3), and gelation by 800 μL of 1.0 M HCl. Moreover, the prepared chitin microgels exhibited pH-dependent swelling-shrinking behavior over a wide range of pH values of between 2–10.

## Figures and Tables

**Table 1:** Parameters in preparation of chitin microgels.

Parameters	Concentration of chitin solution (% *w*/*w*)	O:W volume ratio	HLB of surfactant	Concentration of Span 80 (% *w*/*w*)	Concentration of HCl (M)	Volume of HCl (μl)
Experiment 1	**1** **2** **3**	15:1	4.3	5	1.0	800
Experiment 2	3	**3:1** **7:1** **15:1**	4.3	5	1.0	800
Experiment 3	3	15:1	**4.3** **5** **6**	5	1.0	800
Experiment 4	3	15:1	4.3	**3** **5** **7**	1.0	800
Experiment 5	3	15:1	4.3	5	**0.05** **0.1** **1.0**	800
Experiment 6	3	15:1	4.3	5	1.0	**400** **800** **1200**

**Table 2:** Elemental composition of crab shell, extracted chitin, commercial chitin, and commercial chitosan analyzed by X-ray fluorescence spectroscopy (XRF).

Elements	Crab Shell (mass%)	Extracted Chitin (mass%)	Commercial Chitin (mass%)	Commercial Chitosan (mass%)
C	29.2	52.6	52.2	50.8
O	46.3	47.2	47.6	49.1
Na	0.968	trace	trace	trace
Mg	1.19	trace	trace	0.0135
P	2.93	0.0293	0.0022	0.0074
S	0.344	0.0158	0.0123	0.0044
Cl	0.971	0.0167	0.112	trace
Ca	17.2	0.0782	0.0072	0.0457
Fe	0.016	0.0104	0.0053	0.0084

**Table 3:** Crystallinity index (%CrI), degree of acetylation (%DA), and peak temperature of extracted chitin, commercial chitin, and commercial chitosan.

Samples	%CrI	%DA from XRD	%DA from FTIR	Peak Temperature (°C)
Extracted chitin	70.3	55.3	60.9	330
Commercial chitin	74.7	61.1	62.6	340
Commercial chitosan	44.7	21.2	45.7	295
